# Factors Promoting Learning With a Web Application on Earthquake-Related Emotional Preparedness in Primary School

**DOI:** 10.3389/fpsyg.2020.00621

**Published:** 2020-04-24

**Authors:** Daniela Raccanello, Giada Vicentini, Elena Florit, Roberto Burro

**Affiliations:** ^1^Department of Human Sciences, University of Verona, Verona, Italy; ^2^Department of Developmental Psychology and Socialization, University of Padua, Padua, Italy

**Keywords:** emotional preparedness, earthquake, primary school, achievement emotions, text comprehension, self-concept, web application

## Abstract

Little is known about interventions aimed at building children’s emotional resilience to combat the psychological trauma associated with future earthquakes. However, natural disasters have potentially a highly traumatic impact on children’s psychological functioning. Therefore, within the Emotional Prevention and Earthquakes in a primary school project, we developed a web application promoting earthquake-related emotional preparedness called HEMOT^®^ (Helmet for EMOTions). We studied the role of achievement emotions as factors associated to learning using the web application, coherently with the assumptions of the control-value theory. We also took into account class level and gender. We involved 64 second and fourth graders who used a nine-level web application focused on earthquake-related knowledge, emotions, and emotion regulation. We assessed children’s digital self-concept, learning at the web application (operationalized as digital text comprehension), application-related achievement emotions, and text comprehension. We analyzed the data through linear and generalized linear models, and path analyses. First, our findings documented class differences in some of the examined constructs: Pride was higher for younger compared to older children, while the digital performance was higher for older compared to younger students. Second, digital self-concept was positively linked to application-related pride and relaxation. In turn, pride, relaxation, and sadness, and also text comprehension, were linked to the digital performance. With some exceptions, these relations were in line with the assumptions of the control-value theory, extending it to a context disregarded within the current literature. This knowledge is a first step to develop further interventions fostering children’s resources to promote learning related to emotional preparedness.

## Introduction

Natural disasters, including earthquakes, may have a highly traumatic impact on psychological functioning, both for primary victims experiencing the events directly and for secondary victims indirectly affected through media exposure (Galambos, 2005; Neria et al., 2008; Masten and Osofsky, 2010; Fergusson and Boden, 2014). This is notably true for children, whose vulnerability depends on their level of cognitive and emotional development (Kar, 2009). Beyond long-term traumatic consequences, disturbances in psychological functioning can cause maladaptive behaviors and emotional reactions also during and right away after the emergency phase of a disaster. Nevertheless, prior knowledge on hazardous situations and associated emotions promotes actions mitigating the damage caused by natural disasters, at least in adults (e.g., Hurnen and McClure, 1997; Tekeli-Yeşil et al., 2010).

Several studies focused on psychological interventions following natural disasters with children (Kar, 2009; Pfefferbaum et al., 2019), but little is known about interventions aimed at building children’s emotional resilience to combat the psychological trauma associated with future earthquakes. Therefore, within a larger project (Emotional Prevention and Earthquakes in primary school project, PrEmT project, in Italian Prevenzione Emotiva e Terremoti nella scuola primaria; Raccanello et al., 2019b, 2020; Vicentini et al., 2019), we developed a web application called HEMOT^®^ (Helmet for EMOTions, patent pending; Raccanello et al., 2020)^1^, promoting earthquake-related knowledge, emotions, and emotion regulation.

A recent systematic review (Raccanello et al., 2020) revealed the existence of a number of applications aiming to enhance people’s knowledge on earthquakes, referring to earthquake characteristics and/or safety behaviors. Some applications promote game-based learning and include interactive games, while others are based on lecture-based learning and present written information, oral contents, or sounds typical of earthquakes. Only very rarely, and incidentally, they include elements pertaining to emotional competence, in terms of emotion expression, understanding, and/or regulation (Denham, 1998). Recent studies document that novel technologies and games play an increasing role in teaching, assuming that game-based learning effectiveness is due mainly to the game effect (e.g., Qian and Clark, 2016); however, the role of a larger variety of factors responsible for students’ achievement has not been examined yet.

The efficacy of socio-emotional trainings for children’s emotions has been demonstrated (Durlak et al., 2011), also for trainings on preparedness toward earthquakes (Raccanello et al., 2019b, 2020). However, scarce attention has been paid at the relevance of emotional factors promoting children’s learning through web applications. Among the factors that influence learning, a key role is played by achievement emotions, which are defined as those emotions that students feel in relation to learning activities or outcomes—according to the control-value theory (CVT) (Pekrun, 2006, 2018; Pekrun and Perry, 2014). Achievement emotions can be described by at least two underlying dimensions, valence (positive, negative emotions) and activation (activating, deactivating emotions). According to the CVT, control appraisals such as self-concept beliefs are core determinants of emotions. Control appraisals refer to a learner’s perceived causal influence over one’s actions and outcomes. Earlier research on self-concept emphasized the global nature of self-concept, but the salience of children’s distinction between activities fostered researchers to propose multidimensional models according to which global self-concept can be divided into academic and non-academic components, in turn further divided into subdomain—e.g., related to specific school subjects on the one hand, and physical, social, and emotional domains on the other (Bong and Skaalvik, 2003). According to the CVT, achievement emotions, in turn, influence performance: Positive activating emotions (such as pride) are related positively to achievement, while negative deactivating emotions (such as sadness) are usually related negatively. The pattern is more complex for positive deactivating emotions (such as relaxation) and negative activating emotions (such as anxiety), but overall findings yield that positive emotions are positively linked and negative emotions are negatively linked with performance (Pekrun et al., 2017). Moreover, the CVT postulates that achievement emotions are domain-specific, and several studies have documented this (Pekrun and Linnenbrink-Garcia, 2014; Pekrun, 2018). Rarely the role of emotions relating to the use of e-learning environments has been examined. As an exception, some studies with university students indicated that anxiety can influence perceived satisfaction, in turn impacting self-regulation (e.g., Liaw and Huang, 2013).

Only a few studies assessed achievement emotions in primary school (for exceptions, see Lichtenfeld et al., 2012; Raccanello et al., 2013, 2019a), and to our knowledge, none of them investigated emotions felt during educational trainings, which used technological devices. Examining class level differences, some studies documented higher levels of enjoyment and lower levels of boredom and anxiety for second compared to fourth graders in school domains such as mathematics or native language (Lichtenfeld et al., 2012; Raccanello et al., 2013, 2019a). These findings are in line with the results from longitudinal research reporting a decline in motivation in the transition from primary to secondary school (e.g., Bong, 2009; Wigfield and Eccles, 2000). Concerning self-concept, previous studies suggest that children have unrealistic beliefs on their abilities at the beginning of primary school (Wigfield and Eccles, 2000; Aunola et al., 2002). As they gradually become more able to distinguish ability from effort, and learn to differentiate between ability domains, such beliefs become more realistic but also more negative, and the stability of inter-individual differences increases; this already happens during primary school years (Aunola et al., 2002; Dweck, 2002; Jacobs et al., 2002). In addition, different studies reported gender differences for emotions such as enjoyment, anxiety, or boredom in mathematics or native language, but findings are not consistent (Lichtenfeld et al., 2012; Harari et al., 2013; Lohbeck et al., 2016; Raccanello et al., 2019a).

Finally, still little is known on the role of complex cognitive abilities such as text comprehension for digital tasks. A huge number of studies have defined traditional (i.e., printed) text comprehension as a complex skill, crucial for learning, that develops from preschool onward and is affected by a range of individual cognitive and affective abilities (Oakhill and Cain, 2012; Bråten et al., 2014; Florit et al., 2014). On the contrary, digital text comprehension has been rarely defined (e.g., Singer and Alexander, 2017; Delgado et al., 2018) and has been assessed using a variety of tasks (e.g., linear texts, hypertexts, multiple texts, and complex scenarios; e.g., Mason et al., 2014; Fajardo et al., 2016; LaRusso et al., 2016; Fesel et al., 2018). In addition, less is known on possible changes relating to digital text comprehension abilities during primary school and on the role of individual differences in digital tasks. To date, some studies have analyzed changes in digital text comprehension depending on students’ educational level or cognitive abilities in upper primary and lower secondary school students (e.g., Fajardo et al., 2016; LaRusso et al., 2016). These studies showed that digital text comprehension ability increases in the transition from upper primary to secondary school (LaRusso et al., 2016). Nevertheless, an analysis of changes in digital text comprehension during early primary school is still missing. Finally, comprehension of traditional texts accounted for performance in digital tasks (Fajardo et al., 2016). This evidence, along with findings showing that cognitive abilities are crucial factors in the interplay between emotions and learning (Pekrun and Perry, 2014; Pekrun, 2018), suggests that traditional text comprehension might play a role in digital text comprehension of younger primary school students.

### Aims

We aimed at studying the role of emotions as factors associated to children’s learning within an educational web application on earthquake-related emotional preparedness, HEMOT^®^, coherently with the assumptions of the CVT.

Preliminarily, we examined whether the psychological constructs relating to the digital task—namely digital self-concept, application-related achievement emotions (pride, relaxation, anxiety, and sadness), and digital text comprehension—varied according to class level and gender (Aim 1). On the basis of the literature reporting detrimental motivational and emotional trends (Wigfield and Eccles, 2000; Aunola et al., 2002; Dweck, 2002; Jacobs et al., 2002; Bong, 2009; Lichtenfeld et al., 2012; Raccanello et al., 2013, 2019a), we expected lower scores for digital self-concept and positive achievement emotions for older students compared to younger students, and vice versa for negative emotions. Concerning text comprehension (LaRusso et al., 2016), for older students, we also expected a better performance in the digital task (Hypothesis 1). As regards gender, we did not formulate any specific hypothesis, given previous inconsistent results (Lichtenfeld et al., 2012; Harari et al., 2013; Lohbeck et al., 2016; Raccanello et al., 2019a).

As the main aim (Aim 2), we investigated whether the assumptions of the CVT could be generalized to the processes involved in learning through an educational web application. We explored the predictive role of achievement emotions and text comprehension on the performance at the web application. Coherently with the CVT, we expected positive emotions to be linked positively to performance, and negative emotions to be linked negatively (Hypothesis 2). We also expected text comprehension to be positively linked to performance (Hypothesis 3). Moreover, we hypothesized digital self-concept to be related positively to positive achievement emotions and negatively to negative achievement emotions (Hypothesis 4). Finally, we expected these relations to be equal across class level and gender (Hypothesis 5).

## Method

### Participants

We involved a convenience sample of 64 children attending the second grade and the fourth grade of a primary school in Northern Italy (Table 1). The students had various socioeconomic backgrounds. The parents signed the informed consent form for the participation to the research of their children, and they also completed a questionnaire on children’s sociodemographic data and experience of earthquakes. Most of the children had never experienced earthquakes directly (71%), and only a small percentage of children had experienced them, with no damage (23%). The study was approved by the Ethical Committee of the Department of Human Sciences of University of Verona (protocol n. 134535).

**TABLE 1:** Characteristics of the sample.

**Class level**	***n***	**Mean age**	**Range**	**% Females**	**% Males**
Second grade	28	7.66	7.16–8.10	42%	58%
Fourth grade	36	9.63	9.14–10.04	54%	46%

### Instruments

#### Digital Self-Concept

We adapted a measure previously used to assess domain-specific self-concept (e.g., within a project on the Italian validation of the Achievement Emotions Questionnaire–Elementary School, AEQ-ES, Raccanello et al., 2019a). We administered four items to be evaluated on a five-point scale (1 = *not at all*, 5 = *very much*), referred to the use of tablets and mobile phones (e.g., *I’m good at using mobile phones and tablets*). One item was reversed.

#### Digital Text Comprehension

We operationalized children’s ability to understand digital texts in terms of their performance at HEMOT^®^ (Raccanello et al., 2020), a web application developed *ad hoc*. The digital task consisted in reading written information, supported by a digital voice and accompanied by images or sounds. In developing the contents of HEMOT^®^, we followed suggestions from the design literature (e.g., Brom et al., 2018). Aiming at keeping low the extraneous cognitive load in favor of the intrinsic cognitive load, we did not raise the number of informational elements, including at the same time elements stimulating emotional engagement in terms of pleasant colors or smooth sharpness of edges. HEMOT^®^ includes nine content levels and a final summary level on earthquake-related geological and emotional contents. Each level comprises items (ranging from 24 to 48) formed by a sentence and a drawing (or a sound for six items in level 1), to be evaluated through a dichotomic response, e.g., yes/no (0 = *wrong answer*, 1 = *right answer*). The total maximum score was 296. The nine content levels focus on: (1) nature of earthquakes; (2) safety behaviors; (3) recognition of basic emotions; (4) use of emotional lexicon; (5) earthquake-related emotions; (6) intensity of emotions; (7) intrapersonal and interpersonal emotion regulation strategies; (8) strategies to be effectively used during earthquakes; and (9) strategies to be effectively used after earthquakes (Table 2). Students used a tablet with headphones, with a voice reading written contents to reduce the cognitive load of the reading task.

**TABLE 2:** Examples of items including drawings for the nine levels of HEMOT^®^.

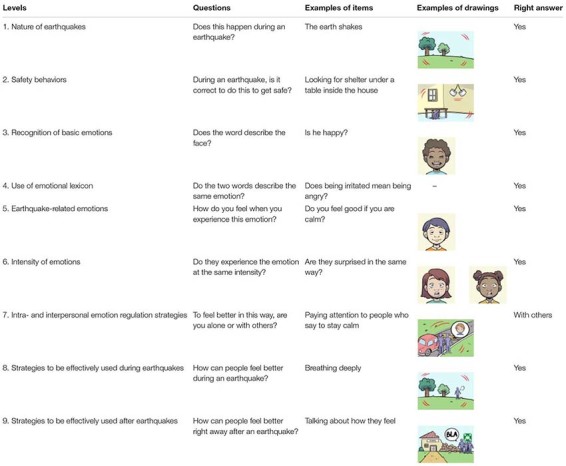

#### Application-Related Achievement Emotions

At the end of each level of HEMOT^®^, we assessed how the children had felt in terms of four achievement emotions, one for each type of Pekrun’s taxonomy (2006): pride as a positive-activating emotion; relaxation as a positive-deactivating emotion; anxiety as a negative-activating emotion; and sadness as a negative-deactivating emotion. We used a short version of the Achievement Emotions Adjective List (AEAL; Brondino et al., 2014; Raccanello, 2015; Raccanello et al., 2014, 2015a,b, 2016). As in previous research (Raccanello et al., 2013, 2017), we used a single-item version, for both time and attentional constraints, presenting one adjective for each emotion (e.g., *How much proud/relaxed/anxious/sad did you feel using this app?*). Each adjective had to be rated on a five-point scale (1 = *not at all*, 5 = *very much*).

#### Text Comprehension

We administered a paper-and-pencil comprehension test (MT-3-Clinica, Cornoldi and Carretti, 2016) to evaluate children’s abilities to understand and decode a narrative text. The texts varied for second- and fourth-grade children and included 12 multiple-choice questions. For each question, the correct answer was coded as 1 and the wrong answer as 0 (total maximum score: 12).

### Procedure

This study refers to the pilot phase of the PrEmT project (Raccanello et al., 2019b, 2020). The data were gathered between February 2019 and May 2019 during regular school hours. The project had the objective to verify the efficacy of a training with activities about knowledge on earthquakes (e.g., characteristics and safety behaviors), emotions (e.g., facial expressions and emotional lexicon), and coping strategies (e.g., emotional regulation strategies useful during and after an earthquake). We used paper-and-pencil tasks to gather measures on digital self-concept and text comprehension, and technological devices (i.e., tablets) to assess digital text comprehension and application-related achievement emotions.

### Data Analysis

We used the R software (R Core Team, 2019) to run linear models and generalized linear models (LMs and GLMs, respectively) and path analyses (PAs). The assumptions of LM and GLM were verified (Warton et al., 2016; Bianchi et al., 2017; Burro et al., 2018).

We carried out five LM and one GLM. In all the models, we included class level (second graders, fourth graders) and gender (males, females) as between-factor fixed effects, scores on digital self-concept, pride, relaxation, anxiety, and sadness as rating dependent variables, and digital text comprehension as a count dependent variable. Respectively, for rating and count variables, we utilized Gaussian family and identity link-function, and Poisson family and log link-function. For the rating variables, we performed an ANOVA table using *F* tests, while for the count variable, we calculated an analysis of deviance table using chi square tests. We calculated effect sizes in terms of Cohen’s *d.* The level of significance was *p* < 0.05.

We conducted a PA to test a model in which digital self-concept related to achievement emotions, in turn linked to digital text comprehension; also text comprehension was linked to digital text comprehension. A preliminary PA indicated that anxiety did not relate significantly to the other variables, and therefore, we excluded it for parsimony. We utilized the SEM function in the lavaan package (Rosseel, 2012), with the Maximum Likelihood with Robust Huber-White standard errors and scaled test statistic (MLR). For running a PA, the minimum ratio between the number of observations and the number of parameters should be 5:1 or more, and preferably 10:1 (Kline, 2016). In our case, the ratio was 6:1, and therefore, the sample size resulted adequate. Given that the variables included in the PA had different ranges, we standardized all the scores before running it. Finally, we ran two multigroup PAs checking the moderating effect of class level and gender. See Table 3 for descriptive statistics and intercorrelations among the variables at issue.

**TABLE 3:** Intercorrelations, descriptive statistics (Means, *M*; Standard Deviations, *SD*; 95% Confidence Intervals, *CI*) for digital self-concept, digital text comprehension, application-related achievement emotions, and text comprehension.

**Variable**	**1**	**2**	**3**	**4**	**5**	**6**	**7**
1. Digital self-concept	−	–0.046	0.365**	0.321*	–0.043	–0.013	−0.310*
2. Digital text comprehension		−	−0.365**	0.030	−0.274*	−0.428**	0.474**
3. Pride			−	0.576**	0.028	0.035	−0.371**
4. Relaxation				−	−0.259*	–0.186	–0.092
5. Anxiety					−	0.751**	–0.069
6. Sadness						−	–0.172
7. Text comprehension							−
*M*	3.87	239.20	2.97	3.55	1.57	1.28	8.00
*SD*	0.95	22.75	1.26	0.99	0.78	0.47	2.24
*95% CI*	0.48	11.37	0.63	0.49	0.39	0.23	1.13

*p < 0.05, **p < 0.01.

## Results

### Linear Models and Generalized Linear Models (Aim 1)

First, the LM on digital self-concept revealed no differences related to class level (second graders: *M* = 3.76; *SD* = 1.18; *CI* = 0.46; fourth graders: *M* = 3.96; *SD* = 0.73; *CI* = 0.25).

Second, the four LMs on achievement emotions yielded a significant effect of class level for pride, *F*(1,60) = 11.32, *p* = 0.001, *d* = 0.68, higher for younger (*M* = 3.51; *SD* = 1.12; *CI* = 0.43) compared to older students (*M* = 2.54; *SD* = 1.20; *CI* = 0.41). No differences emerged for relaxation (second graders: *M* = 3.72; *SD* = 0.99; *CI* = 0.38; fourth graders: *M* = 3.42; *SD* = 0.97; *CI* = 0.33), anxiety (second graders: *M* = 1.71; *SD* = 0.95; *CI* = 0.37; fourth graders: *M* = 1.46; *SD* = 0.60; *CI* = 0.20), and sadness (second graders: *M* = 1.30; *SD* = 0.49; *CI* = 0.19; fourth graders: *M* = 1.27; *SD* = 0.45; *CI* = 0.15).

Third, from the GLM on digital text comprehension, a significant effect of class level emerged, χ*^2^*(1, *N* = 64) = 5.26, *p* = 0.020, *d* = 0.40: Scores were lower for second graders (*M* = 233.57; *SD* = 23.40; *CI* = 9.07) compared to fourth graders (*M* = 243.58; *SD* = 21.55; *CI* = 7.29).

Finally, no gender differences emerged.

### Path Analysis (Aim 2)

**FIGURE 1:**
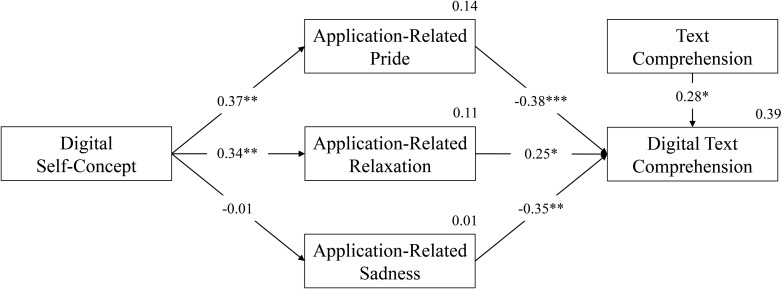
Path analysis for relations between digital self-concept, digital text comprehension, application-related achievement emotions, and text comprehension. We reported explained variances next to each dependent variable. *p < 0.05, **p < 0.01, ***p < 0.001.

The PA (Figure 1) indicated that digital self-concept was positively linked to application-related pride (β = 0.37, *p* = 0.001) and relaxation (β = 0.34, *p* = 0.007), explaining, respectively 14% and 11% of the variance. In turn, the three achievement emotions related significantly to digital text comprehension, positively for relaxation (β = 0.25, *p* = 0.025) and negatively for pride (β = −0.38, *p* < 0.001) and sadness (β = −0.35, *p* = 0.006). Finally, text comprehension was positively related to digital text comprehension (β = 0.28, *p* = 0.015). Explained variance for digital text comprehension was 39%.

Finally, we conducted two multigroup PAs to check whether class level and gender moderated the described relations. The findings indicated that the tested relations did not change according to the two factors.

## Discussion

Within a project on earthquake-related emotional preparedness, the PrEmT project (Raccanello et al., 2019b, 2020), we studied the role of emotions and their antecedents as factors associated to children’s learning through an educational web application. The web application promotes awareness on safety procedures and earthquake-related emotions and emotion regulation strategies. Its use can enhance children’s preparedness and resilience, helping to reduce both physical and psychological impairments caused by possible natural disasters such as earthquakes. Our findings enabled us to generalize the assumptions of the CVT of achievement emotions to this context, filling a gap in current psychological literature.

As a preliminary aim, we examined class level and gender differences in digital self-concept, application-related achievement emotions, and digital text comprehension. Concerning class level, our findings indicated that second graders did not differ so markedly from fourth graders. While the literature on emotions and motivation for learning has usually documented a detrimental trend at increasing ages (Wigfield and Eccles, 2000; Bong, 2009; Lichtenfeld et al., 2012; Raccanello et al., 2013, 2019a), our analyses did not yield any class level difference for digital self-concept, and only for pride among application-related achievement emotions. We could speculate that the absence of a marked worsening could be due to a variety of factors. First, our findings indicated the stability of digital self-concept between the two ages, giving new data on a scarcely examined issue, that should be confirmed in future studies. Second, the two groups differed only for a reduced age range. Third, usually in the traditional school contexts, the tasks and the contents to be mastered become objectively more difficult as time goes by, probably fostering the described detrimental trend. However, these changes did not characterize the digital tasks included in the HEMOT^®^ application. Fourth, both data on the higher intensity of positive emotions compared to negative emotions (emerging from the exam of the means of the achievement emotions) and anectodical information gathered on how much the children liked the application clearly indicated that the digital task was perceived on the whole as very attractive, differently from what usually happens in relation to school subjects. Finally, class level differences characterized nonetheless digital text comprehension, higher for older students, extending research on digital text comprehension in older students (LaRusso et al., 2016). Moreover, it is worth noting that these data are a first step to further develop the HEMOT^®^ application including score thresholds to have access to the following level, according to users’ age.

Comparing males and females, our analyses revealed no differences, in particular for digital text comprehension. In the literature, gender differences in performance are explained resorting to several possible factors, such as differences in cognitive skills, gender identity, motivation, or gender stereotypes (Freedman-Doan et al., 2000; Cvencek et al., 2011; Steffens and Jelenec, 2011; Martinot et al., 2012). On the whole, such factors would suggest an advantage of females for reading and an advantage of males for technology-related learning (e.g., Steffens and Jelenec, 2011). However, these issues have been explored mainly with older students. In our case, we did not specifically assess constructs related to the above-mentioned four factors. Nevertheless, we could speculate that the absence of gender differences could be linked to a confounding due to the effects of these two opposite tendencies (i.e., females outperforming males for reading, and vice versa for technology-related learning) in a task in which abilities relating both to comprehension and to the use of technological devices are highly salient to determine students’ performance. Future studies exploring systematically the role of the four different factors could help to disambiguate these findings.

Documenting class level and gender differences was a first step to explore the nature of the factors associated with a good performance in educational applications such as HEMOT^®^. A second and key step was to explore whether application-related achievement emotions and their antecedents were linked to learning as assumed by the CVT, as further resources helping to promote learning. Our findings indicated that the digital self-concept was positively linked to pride and relaxation. In turn, pride, relaxation, and sadness were linked to the digital performance, i.e., positively for relaxation and negatively for pride and sadness. With the exception of the relation between pride and digital text comprehension, these relations were in line with the assumptions of the CVT, extending it to a context disregarded within the current literature. The exception for pride could be due to the fact that the students were asked to report it within a task that did not include any feedback on their actual performance. Therefore, the absence of information on the accuracy of their responses could have provoked a false perception on one’s own performance, overestimating it also when it was not excellent. It is worth noting that anxiety did not play a relevant role in the examined context. Notwithstanding the predominant role of this emotion for learning in a variety of school contexts (e.g., Zeidner, 1998, 2014), our data suggest that for technological tasks such as the one proposed through the HEMOT^®^ application, its influence would be not so detrimental. Further research should examine deeper possible underlying reasons.

Our study suffers from limitations related to the self-report nature of some of the instruments used, such as social desirability. However, such methods have the great advantage of enabling direct access to people’s inner states, and they are still among the privileged ways to explore people’s representation of one phenomenon (Pekrun and Bühner, 2014). In addition, we piloted the web application involving only a small sample. Future research will use the HEMOT^®^ application (Raccanello et al., 2020) with larger groups, also testing its feasibility with older students. The final version of HEMOT^®^ will be available via https://www.hemot.eu. Moreover, we did not examine the role of previous knowledge on the topics included within the web application. Furthermore, we did not check whether the training impacted students’ self-concept, as documented for example for self-efficacy in training using Massive Open Online Courses (MOOC; García-Martín and García-Sánchez, 2020); future research could investigate this issue. Finally, we focused only on earthquakes. It would be interesting to adapt the HEMOT^®^ application for a larger range of disasters, both natural and technological, as a step to support awareness of people for fostering their resilience.

To sum up, our findings contribute to increasing the theoretical knowledge on factors fostering learning through an educational web application on earthquake-related emotional prevention, extending the generalizability of the CVT to this peculiar context and giving hints for fostering the efficacy of future intervention programs.

## Data Availability Statement

The raw data supporting the conclusion of this article will be made available by the authors to any qualified researcher upon request.

## Ethics Statement

The study involving human participants was reviewed and approved by the Ethical Committee of the Department of Human Sciences of University of Verona (protocol n. 134535). Written informed consent to participate in this study was provided by the participants’ legal guardian/next of kin.

## Author Contributions

DR, GV, EF, and RB contributed to conception and design of the study and wrote the sections of the manuscript. DR, GV, and RB organized the database. RB performed the statistical analysis. DR wrote the first draft of the manuscript. All authors contributed to manuscript revision, read, and approved the submitted version.

## Conflict of Interest

The authors declare that the research was conducted in the absence of any commercial or financial relationships that could be construed as a potential conflict of interest.

## Footnotes

1 https://www.hemot.eu
